# Protein lactylation influences atherosclerotic plaque stability by regulating macrophage functions

**DOI:** 10.3389/fimmu.2026.1789108

**Published:** 2026-03-09

**Authors:** Wenyan Liu, Ruimin Chen, Zonghu Jia, Shufang Han, Qun Jin

**Affiliations:** 1 Department of Cardiology, The 960th Hospital of the Joint Logistics Support Force of the Chinese People’s Liberation Army, Jinan, Shandong, China; 2 Graduate School, Shandong First Medical University and Shandong Academy of Medical Sciences, Jinan, Shandong, China

**Keywords:** coronary atherosclerosis, lactate, macrophage, plaque stability, protein lactylation

## Abstract

Atherosclerosis (AS) is a leading cause of cardiovascular events and mortality worldwide. Plaque stability is a direct determinant of clinical outcomes for patients. Within the hypoxic and inflammatory microenvironment of plaques, glycolysis is significantly enhanced in macrophages and other cells, leading to substantial lactate accumulation. Recent studies reveal that lactate serves not only as a metabolic byproduct but also as a substrate for a novel post-translational modification, driving dynamic reversible protein lactylation. This review systematically demonstrates that protein lactylation acts as a critical molecular bridge, linking cellular metabolic dysregulation to immune-inflammatory responses. It precisely regulates multiple macrophage functions, such as polarization, programmed cell death, and phagocytic efficiency. Through these mechanisms, it profoundly influences key pathological processes. These processes include plaque inflammation and repair, necrotic core formation, and local thrombogenesis. Ultimately, protein lactylation emerges as a pivotal regulatory mechanism governing the stability and evolution of AS plaques. Furthermore, this review explores potential therapeutic strategies targeting this modification network, aiming to advance the clinical translation of related research.

## 1 Introduction

Atherosclerosis (AS) is a chronic, progressive inflammatory disease of the arterial wall (1, 2). The acute cardiovascular events it causes, such as myocardial infarction and ischemic stroke, remain leading causes of death globally (3, 4). According to the World Health Organization (WHO), AS and its complications are responsible for approximately 17 million deaths annually, accounting for 31% of global mortality (5). In coronary atherosclerosis, the stability of the atherosclerotic plaque is a direct determinant of disease progression and clinical outcomes. The rupture of a vulnerable plaque can trigger acute thrombosis (6), which is a critical event leading to clinical syndromes such as acute coronary syndrome (ACS).

Among the various cell types within plaques, macrophages play a central role (7). They phagocytose oxidized low-density lipoprotein (ox-LDL) and transform into foam cells. This process drives lipid deposition and exacerbates local inflammatory responses, thereby profoundly influencing plaque stability (8). Within the hypoxic and inflammatory microenvironment of AS plaques, glycolysis is significantly enhanced, leading to substantial accumulation of lactate (9). Recent studies reveal that lactate is not merely the end product of glycolysis; it also functions as an important signaling molecule in microenvironment regulation (10) and serves as a substrate for protein lactylation modifications (11).

In the context of AS, this novel post-translational modification mediates metabolic reprogramming and functional regulation in macrophages. It influences macrophage polarization, the release of inflammatory cytokines, and efferocytosis, consequently regulating plaque stability (12–14). Notably, protein lactylation acts as a critical metabolic-immune regulatory hub, offering a fresh perspective for systematically understanding AS progression. This review systematically examines the specific mechanisms through which protein lactylation influences coronary atherosclerotic plaque stability by regulating macrophage function. It aims to reveal new pathways of metabolic-immune interaction in AS progression. Furthermore, it seeks to provide a theoretical foundation for developing diagnostic and therapeutic strategies targeting lactylation modifications and to advance related research toward clinical translation.

## 2 Lactate and protein lactylation

Traditionally, lactate has been regarded as a metabolic waste product generated by glycolysis under hypoxic conditions (15). However, in the 1920s, Otto Warburg discovered that cancer cells preferentially convert glucose to lactate, even in the presence of sufficient oxygen. This phenomenon is known as the “Warburg effect” (16). This suggested that lactate production might be an active metabolic choice rather than merely a passive byproduct of hypoxia. With further research, lactate is now recognized not only as an important energy carrier and metabolic substrate but also as a critical signaling molecule. It mediates intercellular communication through specific receptors such as G protein-coupled receptor 81 (GPR81) (17). Notably, lactate can directly serve as an acyl donor, driving a novel post-translational modification known as lactylation, thereby enabling profound regulation of cellular functions at the molecular level.

In 2019, Yingming Zhao’s team (18) first revealed the novel post-translational modification mechanism of histone lysine lactylation. Its biochemical essence is the covalent bonding of a lactyl group from a lactate molecule to the ϵ-amino group of a target protein’s lysine residue, forming lactylated lysine (Kla). This reaction is catalyzed by lactyltransferases (also known as writer enzymes). The modification is widespread in both histones and non-histone proteins (19, 20), and its level is dynamically regulated by lactate concentration. This effectively translates real-time changes in cellular metabolic activity into precise regulation of gene transcription and protein function. In histones (e.g., histone H3 lysine 18 lactylation [H3K18la]), lactylation regulates gene expression by altering local chromatin structure. In non-histone proteins, such as the metabolic enzyme pyruvate kinase M2 (PKM2) and the inflammatory mediator high-mobility group box 1 (HMGB1) (21), lactylation modulates function by affecting protein activity, stability, subcellular localization, and interaction networks. This process is dynamically and reversibly fine-tuned by writer enzymes, such as the histone acetyltransferases p300 and CREB-binding protein (CBP), and by eraser enzymes, including histone deacetylases 1-3 (HDAC1-3) and sirtuins 1-3 (SIRT1-3) (22). This modification thus represents a versatile and dynamic regulatory layer that integrates metabolic information into the control of cellular phenotype and function.

## 3 Metabolism and protein lactylation in AS plaques

### 3.1 Metabolic characteristics of the plaque microenvironment

AS plaques, particularly their lipid core regions rich in immune cells, represent a classic microenvironment of metabolic disorder (23). This area often develops structural hypoxia due to insufficient blood perfusion, which stabilizes hypoxia-inducible factor-1α (HIF-1α) and drives cells to enhance glycolysis (24). Macrophages, the predominant immune cells within plaques, activate and transform into foam cells upon stimulation by ox-LDL (25). This process impairs mitochondrial oxidative phosphorylation while dramatically increasing glycolytic flux. Additionally, vascular smooth muscle cells undergo phenotypic conversion under inflammatory cytokine stimulation (26), and activated endothelial cells (27) also exhibit enhanced glycolytic activity. Collectively, these cells contribute to substantial lactate production and accumulation within plaques.

### 3.2 Direct evidence of lactylation modifications in macrophages within AS plaques

**Figure 1:**
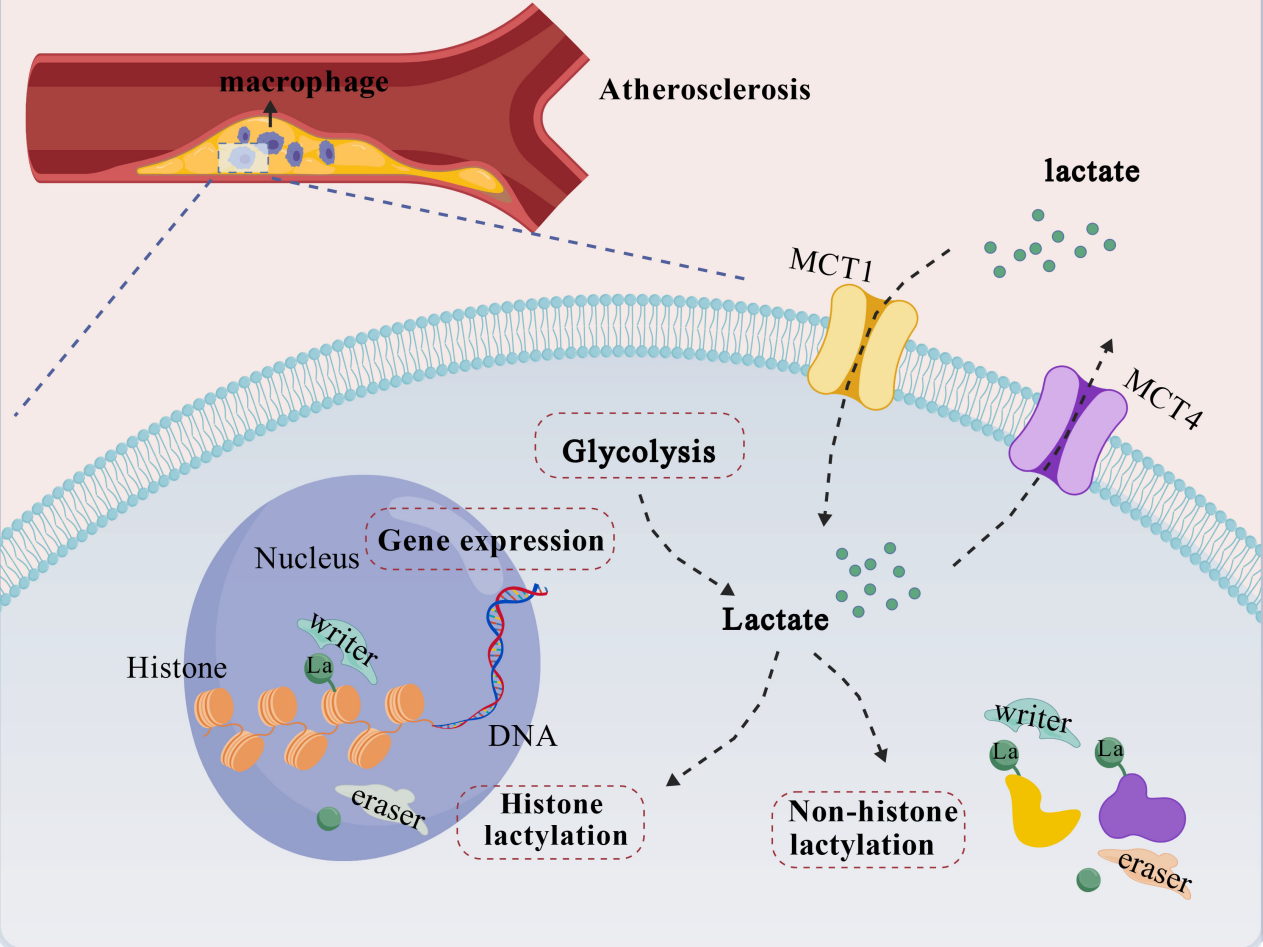
Protein lactylation in macrophages within the atherosclerotic plaque microenvironment. In the atherosclerotic plaque microenvironment, lactate levels are elevated both through enhanced macrophage glycolysis and uptake from the extracellular milieu. Lactate, transported by MCTs, serves as a substrate for nuclear and cytoplasmic protein lactylation. In the nucleus, histone lactylation (e.g., catalyzed by p300/CBP) epigenetically regulates gene expression. In the cytoplasm, non-histone protein lactylation alters their functional properties. This reversible modification, orchestrated by “writer” and “eraser” enzymes, enables macrophages to translate metabolic changes into altered functions that impact plaque fate. Created with BioGDP.com (30).

Multiple studies have directly confirmed the occurrence and functional role of protein lactylation within AS plaques. Bioinformatic analysis reveals that lactylation-related genes are upregulated in AS and show a positive correlation with the degree of macrophage infiltration (28). In the apolipoprotein E knockout (ApoE^−^/^−^) mouse model, immunofluorescence staining demonstrates that various lactylation signals are specifically enriched in the macrophage-infiltrated areas (F4/80^+^) of plaques (29). Monocarboxylate transporters 1 and 4 (MCT1/4) are key proteins responsible for lactate transport. Further interventional studies confirm that inhibiting the lactate transporter MCT4 to increase intra-plaque lactate accumulation can upregulate H3K18la levels. This drives macrophage polarization toward the M2 reparative phenotype and ultimately improves key stability indices such as plaque area, necrotic core size, and collagen content. Collectively, evidence from correlative analyses to functional experiments indicates that protein lactylation is a dynamically present modification within the AS plaque microenvironment. It plays a pivotal role in linking metabolic dysregulation to macrophage functional reprogramming, as summarized in **Figure 1**.

## 4 Protein lactylation regulates macrophage functions in multiple ways

### 4.1 Regulation of macrophage polarization and its impact on AS plaque inflammation

Chronic non-resolving inflammation is the central pathological process driving the initiation, progression, and ultimate destabilization of AS plaques. Inflammation permeates every stage of the lesion, directly linking risk factors such as dyslipidemia to plaque formation (31). In this process, macrophages, serving as the core inflammatory effector and regulatory cells, profoundly shape the plaque microenvironment through the dynamic evolution of their phenotypes and functions (32). In the early stages of the lesion, M2-type macrophages predominate within the plaque. Their metabolism is primarily characterized by oxidative phosphorylation and fatty acid oxidation (33). These cells promote plaque stability by secreting collagen and also enhance the clearance capacity for apoptotic cells (34). As inflammation intensifies, the proportion of M1-type macrophages gradually increases.

Activated M1-type macrophages undergo significant metabolic reprogramming, characterized by enhanced aerobic glycolysis and increased lactate production (35, 36). At this stage, lactate preferentially induces histone H4 lysine 12 lactylation (H4K12la). This modification directly activates the transcription of genes such as lactate dehydrogenase A (LDHA) and HIF-1α, establishing a glycolysis-H4K12la-PKM2 positive feedback loop. This loop collectively drives the substantial release of pro-inflammatory cytokines, including interleukin-1β (IL-1β) and tumor necrosis factor-α (TNF-α), thereby establishing and reinforcing a pro-inflammatory microenvironment (37–39).

As inflammation progresses and lactate continues to accumulate, a temporal regulatory effect of lactylation gradually emerges (40). The accumulated lactate serves as a substrate and, catalyzed by acetyltransferases such as p300/CBP, specifically promotes lactylation at H3K18la (26). This H3K18la modification is specifically induced during the late stages of M1 polarization. Its function is time-dependent, capable of initiating the transcription of specific genes in a programmed manner, thereby promoting tissue repair and the M2 polarization process. H3K18la is specifically enriched in the promoter regions of anti-inflammatory genes (e.g., interleukin-10 [IL-10], transforming growth factor-β [TGF-β]) and tricarboxylic acid (TCA) cycle genes (e.g., pyruvate dehydrogenase E1 subunit alpha 1 [PDHA1], isocitrate dehydrogenase 2 [IDH2]), directly activating their transcription. This drives the transition of macrophages toward the M2 phenotype, with a concomitant metabolic reprogramming from glycolysis back to oxidative phosphorylation. In the ApoE^−^/^−^ mouse model, inhibiting the macrophage lactate efflux transporter MCT4 leads to intracellular lactate accumulation and elevated H3K18la levels. This subsequently promotes M2 polarization and ultimately results in reduced atherosclerotic plaque area, decreased necrotic core ratio, and increased collagen content (29).Concurrently, lactate also exerts negative feedback regulation through non-histone lactylation. Lactate lactylates PKM2 at lysine 62 (K62) (41), thereby stabilizing its highly active tetrameric conformation. This enhances the pyruvate kinase activity of PKM2 in the cytoplasm but simultaneously inhibits its nuclear translocation, reducing its synergistic pro-inflammatory transcriptional activity with HIF-1α.Thus, lactylation modifications finely balance glycolytic metabolic flux and inflammatory signaling output at multiple molecular levels, serving as a core molecular switch regulating the processes of inflammatory damage and repair.

The regulation of macrophage fate by lactylation exhibits fundamental context dependency. In the progression of abdominal aortic aneurysm (42), H3K18la induces the activation of glioma-associated oncogene homolog 3 (GLI3), driving macrophage polarization toward the M1 phenotype. In pathological chronic inflammatory microenvironments, such as those found in diabetes (43) and neurodegenerative diseases (39), the H4K12la-dominated pro-inflammatory pathway may remain persistently activated, hindering inflammation resolution. Conversely, under physiological stress such as exercise, elevated systemic lactate levels promote the specific lactylation of methyl-CpG binding protein 2 (MeCP2) at lysine 271 (K271) in macrophages (44). This modification drives macrophage polarization toward the M2 phenotype by inhibiting the pro-inflammatory transcription factor runt-related transcription factor 1 (RUNX1), consequently conferring protective effects in animal models. These effects include reduced plaque burden, increased collagen content, and improved lipid profiles. Together, these findings confirm that lactylation-mediated regulation of macrophage polarization exhibits both temporal specificity and context dependency. Its functional output may be subject to complex influences from the local cytokine milieu, the duration of lactate exposure, and its interplay with other epigenetic modifications. At present, a unified framework is still lacking to predict which polarization outcome lactylation will predominantly drive at different stages of AS or within distinct microenvironmental niches.

However, it must be pointed out that the aforementioned classic framework based on the M1/M2 dichotomy, while helpful for initially elucidating the regulatory logic of lactylation, is no longer sufficient to fully reflect the true complexity of macrophages within atherosclerotic plaques. Fine-resolution single-cell transcriptomic analyses have revealed that macrophages in plaques are highly heterogeneous, including but not limited to: lipid-associated TREM2^hi^ macrophages, pro-inflammatory macrophages, proliferative macrophages, and other subsets with distinct transcriptional states. These subsets exhibit significant differences in spatial distribution, metabolic characteristics, and functional output. Therefore, the regulation of macrophages by lactylation modification likely does not simply follow the M1-M2 axis but differentially influences specific functional subsets. For example, does lactylation preferentially regulate the lipid metabolism and inflammatory balance of TREM2^hi^ macrophages? Does it affect the self-renewal capacity of proliferative macrophages? Current research has not yet been conducted at this finer cellular resolution. Placing lactylation research within such a modern cellular atlas that transcends the dichotomy is key to understanding its pathophysiological significance.

### 4.2 Regulation of cell death and efferocytosis affecting the necrotic core of AS plaques

The death of macrophages and the failure of efferocytosis lead to the continuous accumulation of cellular debris and lipid contents that cannot be cleared, forming the characteristic atheromatous necrotic core. The expansion of this core, coupled with an active inflammatory environment, progressively weakens the plaque’s fibrous cap, directly driving the plaque toward an unstable and rupture-prone state (45). Within AS plaques, the induction of macrophage ferroptosis constitutes a significant pathway through which lactylation drives necrotic core formation. Studies have confirmed (46) that the high-lactate environment within plaques drives H3K18la, a process catalyzed by p300. This modification directly upregulates the expression of the methyltransferase-like 3 (METTL3) enzyme, which is responsible for N6-methyladenosine (m^6^A) RNA modification. METTL3, in turn, mediates m^6^A modification of the solute carrier family 7 member 11 (SLC7A11) mRNA, which encodes the cystine/glutamate antiporter. This modification leads to the recognition and degradation of SLC7A11 mRNA by the YTH N6-methyladenosine RNA binding protein F2 (YTHDF2), thereby inhibiting glutathione synthesis and triggering lipid peroxidation and ferroptosis. Macrophages undergoing ferroptosis release oxidized lipids and pro-inflammatory factors, directly contributing to the inflammation and expansion of the necrotic core.

Additionally, lactylation modifications are also implicated in regulating macrophage pyroptosis. *In vitro* experiments have demonstrated that exogenous lactate elevates global protein lactylation levels in THP-1-derived macrophages in a dose-dependent manner, concurrently activating NLRP3 inflammasome-dependent pyroptosis and the secretion of pro-inflammatory cytokines (47). In chronic inflammatory diseases such as atherosclerosis, vascular smooth muscle cells (VSMCs) can transdifferentiate into macrophage-like cells with pro-inflammatory properties, driving vascular pathology and complications. Research has shown that under stimulation by inflammatory signals such as TNF-α, the intracellular PI3K/AKT/ERK signaling pathway is activated. This leads to phosphorylation at serine 24 (S24) of the Sox10 protein, which is further modified by lactylation. Together, these modifications confer full transcriptional activity to Sox10 (48). The activated Sox10 drives VSMC reprogramming, inducing the expression of a set of canonical macrophage functional genes (such as Cd74, C3, and Lyz2), while simultaneously upregulating pyroptosis execution proteins including GSDMD and caspase-1. This cascade promotes inflammatory death in VSMCs and amplifies vascular inflammation through the release of additional inflammatory mediators. This mechanism illustrates that lactylation modifications not only directly affect macrophages but can also indirectly regulate inflammatory and cell death processes—and thereby plaque stability—by controlling the transdifferentiation of other cell types (e.g., VSMCs) into “macrophage-like” phenotypes. Inhibition of the PI3K/AKT pathway, and consequently Sox10, via RGSS alleviates vascular inflammation. Furthermore, studies in liver injury models have confirmed that lactylation at lysine 33 (K33) of the NEDD4 ubiquitin ligase directly impairs its binding to caspase-11. This results in the loss of NEDD4-mediated ubiquitinative inhibition of caspase-11 (49). The activated caspase-11 then cleaves GSDMD, triggering the non-canonical pyroptosis pathway.

Beyond promoting cell death, lactylation can also impair the cleanup capacity of macrophages. H4K12la directly activates the transcription of ADAM17. Upregulated ADAM17 cleaves the extracellular domain of the phagocytic receptor MerTK, thereby depriving macrophages of their ability to recognize and engulf apoptotic cells and leading to a significant decline in efferocytosis efficiency (50). This series of cellular events, meticulously regulated by protein lactylation, collectively contributes to a substantially increased risk of atherosclerotic plaque rupture.

### 4.3 Regulation of thrombotic propensity in AS

Secondary thrombosis following the rupture of an AS plaque is the direct cause of clinical events such as ACS. Protein lactylation not only indirectly influences plaque rupture risk by remodeling plaque structure but also directly regulates the local pro-thrombotic microenvironment within the plaque.This effect is primarily achieved by modifying key mediators that influence platelet activation, which is a central event in thrombosis. The high-lactate microenvironment within plaques can significantly alter the secretory profile of macrophages. Stimulated by lactate, HMGB1 protein in macrophages undergoes dual lactylation and acetylation modifications. This post-translational modification not only enhances the active secretion of HMGB1 but also prolongs its extracellular half-life. HMGB1 released into the local plaque or circulation acts as a potent damage-associated molecular pattern (DAMP). It can bind to Toll-like receptor 4 (TLR4) on the platelet surface, activating its downstream signaling cascade. This cascade involves the adapter protein myeloid differentiation primary response 88 (MyD88) and culminates in the activation of the transcription factor nuclear factor kappa-light-chain-enhancer of activated B cells (NF-κB). Activation of this pathway induces the expression of P-selectin (CD62P) on the platelet surface, promotes the formation of platelet-leukocyte aggregates (PLAs), and initiates the coagulation cascade, thereby significantly increasing thrombotic propensity (21, 51, 52). Further experiments indicate that specifically inhibiting the lactylation of HMGB1 can effectively reduce the formation of PLAs in both the circulation and within plaques, and decrease the microthrombus burden in plaques. This directly proves that the lactate-HMGB1 lactylation-platelet activation axis is a key pathological bridge connecting metabolic dysregulation, inflammation, and acute thrombotic events. It provides a highly promising novel target for preventing fatal thrombosis following plaque rupture.

**Figure 2:**
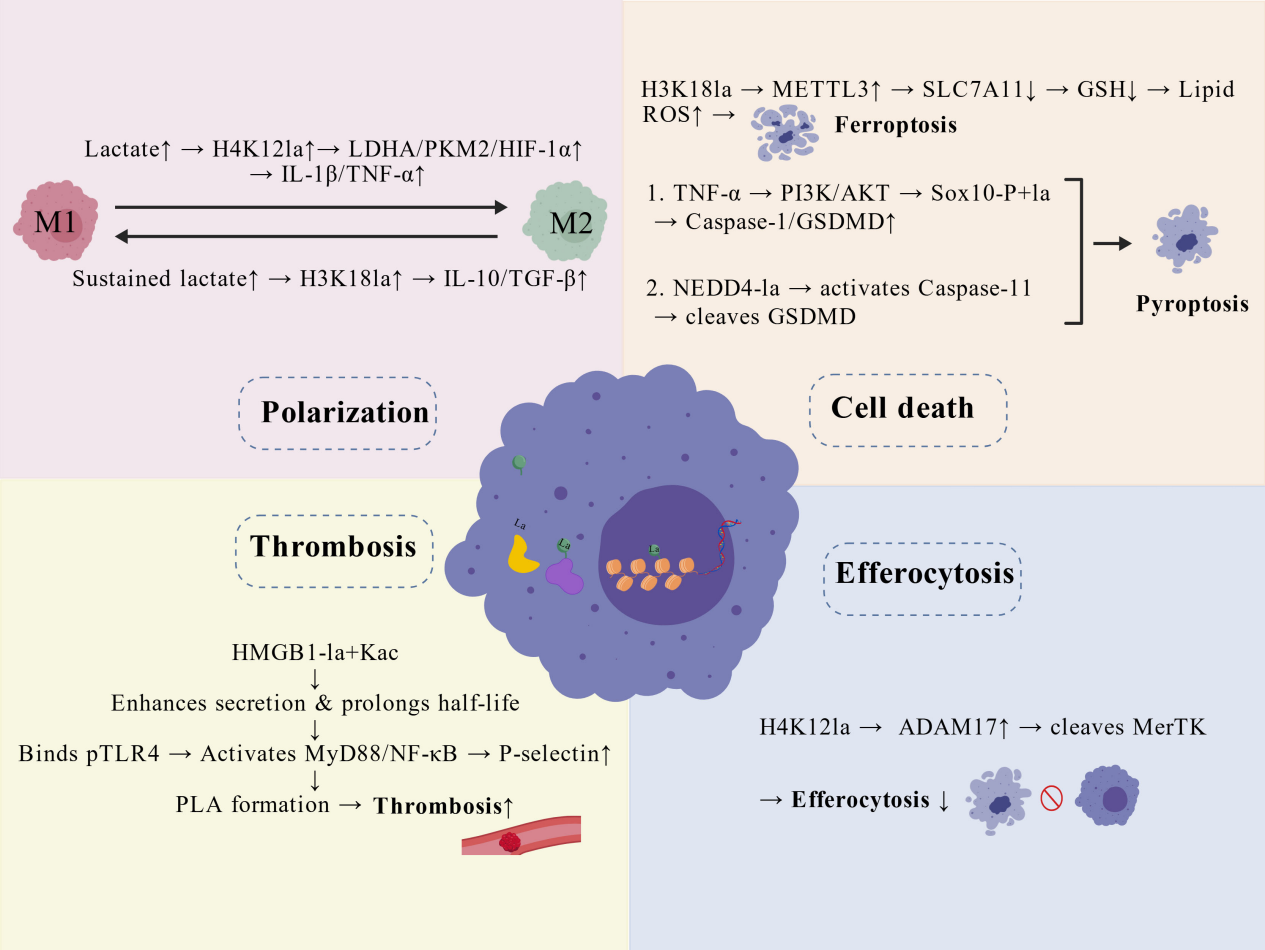
Regulation of macrophage functions by protein lactylation in atherosclerosis. Elevated plaque lactate drives macrophage responses via specific lactylation marks. Polarization: H4K12la promotes M1 pro-inflammatory signaling (IL-1β/TNF-α), while sustained lactate-induced H3K18la shifts cells toward an M2 reparative phenotype (IL-10/TGF-β). Thrombosis: Dual-modified (La+Kac) HMGB1 enhances secretion, activates platelet TLR4/MyD88/NF-κB, and increases P-selectin/PLA formation. Cell Death: H3K18la upregulates METTL3, leading to SLC7A11 loss, glutathione depletion, and ferroptosis. Lactylation also promotes pyroptosis via Sox10-P+la (Caspase-1/GSDMD↑) and via NEDD4-la-mediated Caspase-11 activation. Efferocytosis: H4K12la increases ADAM17, which cleaves MerTK, impairing apoptotic-cell clearance. Created with BioGDP.com (30).

The multifaceted regulation of macrophage functions by protein lactylation, encompassing polarization, cell death, efferocytosis, and thrombotic propensity, is schematically summarized in **Figure 2**. However, in summary, the mechanistic evidence chain linking lactylation to the regulation of macrophage death, efferocytosis, and thrombotic propensity remains incomplete. A significant limitation is that many key findings originate from *in vitro* cell models using “dose-dependent” experimental designs. While such approaches can reveal mechanistic associations, the lactate stimulation concentrations and patterns employed often do not accurately correspond to the complex, dynamic pathophysiological range within atherosclerotic plaques. More importantly, the causal directionality between the observed lactylation modifications and functional phenotypes in many studies has not been fully clarified. For example, research has shown that exogenous lactate treatment can simultaneously elevate global protein lactylation levels and trigger pyroptosis. However, such experiments can only demonstrate their co-occurrence and fail to address a fundamental question: Is the observed increase in overall lactylation levels a necessary and sufficient condition driving pyroptosis? In other words, do specific lactylation modifications on particular proteins directly initiate the cell death program (as an upstream driver), or does the metabolic dysregulation triggered by the pyroptotic process lead to widespread protein modification (as a downstream consequence)? Currently, experimental evidence unequivocally confirming the driving causal role of specific lactylation events remains limited.

## 5 Potential therapeutic strategies targeting the protein lactylation regulatory network

### 5.1 Targeting the regulation of lactate homeostasis in the AS plaque microenvironment

Intervening in the balance between lactate production and clearance represents a fundamental strategy for regulating lactylation levels at their source. Firstly, inhibiting excessive lactate production is a direct approach. The application of small-molecule inhibitors targeting LDHA, such as FX-11 and oxamate (53, 54), can effectively reduce the conversion of pyruvate to lactate, thereby lowering intracellular lactate levels. In AS models (55), inhibition of LDHA has been proven to alleviate macrophage inflammation and delay plaque progression. Furthermore, using hexokinase (HK) inhibitors (e.g., 2-deoxy-D-glucose [2-DG]) or pyruvate dehydrogenase kinase (PDK) inhibitors like dichloroacetate can indirectly reduce lactate generation by suppressing glycolytic flux or promoting pyruvate entry into the TCA cycle (56, 57).

Secondly, precise modulation of lactate transport is crucial. MCTs serve as gateways regulating intracellular lactate concentration. As mentioned earlier, in macrophages, using compounds like VB124 to inhibit MCT4 expression can increase intracellular lactate accumulation. This specifically induces H3K18la, thereby activating anti-inflammatory and metabolic reprogramming genes, driving macrophages toward a reparative M2 phenotype, and enhancing plaque stability (29). Non-steroidal anti-inflammatory drugs (NSAIDs) (e.g., diclofenac, indomethacin) and natural products like quercetin have also been found to possess MCT inhibitory activity, offering possibilities for drug repurposing (58, 59).

### 5.2 Targeting writer and eraser enzymes of lactylation modifications

Directly regulating the enzymatic processes of lactylation is central to achieving precise therapeutic intervention. On one hand, inhibiting the activity of writer enzymes can block pathological lactylation. The histone acetyltransferase p300 has been confirmed to possess broad lactyltransferase activity. Developing its specific small-molecule inhibitors (such as C646 and A-485) holds promise for disrupting pathogenic transcriptional programs induced by high-lactate environments (60, 61). Recent research has discovered (62) that alanyl-tRNA synthetase 1 (AARS1) is a novel global lactyltransferase. β-alanine can act as its competitive inhibitor by occupying AARS1’s lactate-binding site, effectively reducing lactylation levels of key proteins like p53. This provides a novel upstream target for atherosclerosis therapy.

On the other hand, activating the function of eraser enzymes may promote a protective phenotype. SIRT1–3 and HDAC1–3 possess deacetylase activity, which may also extend to the removal of lactyl groups. For instance, the SIRT1 agonist resveratrol might promote the expression of anti-inflammatory and reparative genes while eliminating pathogenic lactylation modifications on proteins such as HMGB1 (21). Honokiol, by activating SIRT3 to downregulate lactylation levels, has shown potential protective value in disease models (63).

### 5.3 Targeting key lactylated proteins and their effector pathways

Intervening in core lactylated proteins and their downstream pathways, which have been validated in AS, can make therapeutic strategies more targeted. Research has found (64) that during AS progression, histone lactylation-related genes such as poly(ADP-ribose) polymerase family member 12 (PARP12), integrin subunit beta 2 (ITGB2), acid phosphatase 5 (ACP5), and interferon regulatory factor 8 (IRF8) are significantly enriched in immune- and inflammation-related pathways. These are particularly associated with NOD-, LRR- and pyrin domain-containing protein 3 (NLRP3) inflammasome activation and macrophage pyroptosis. The traditional Chinese medicine formula Ruanjian Qingmai Formula can effectively inhibit macrophage pyroptosis by regulating the expression of these genes, thereby reducing AS plaque area.

### 5.4 Exploring novel mechanisms of existing cardiovascular drugs

The beneficial mechanisms of many clinically effective cardiovascular drugs may partially stem from their regulation of lactate metabolism or the lactylation network, providing insights for drug repurposing. Atorvastatin, a 3-hydroxy-3-methylglutaryl-coenzyme A (HMG-CoA) reductase inhibitor, can influence lactate transport by interfering with the function of MCT1/4, thereby modulating intracellular lactate levels (65). This mechanism not only offers a new perspective for understanding its muscle-related side effects but also provides a novel rationale for developing strategies targeting the lactylation network to intervene in plaque inflammation.

Metformin, a first-line antidiabetic drug, suppresses lactate production by inhibiting the expression of lactate-related genes, including HDAC3, PKM2, and glyceraldehyde-3-phosphate dehydrogenase (GAPDH). This further reduces H3K18la levels, thereby decreasing reactive oxygen species (ROS) generation and exerting anti-inflammatory effects (66). Furthermore, sodium-glucose cotransporter 2 (SGLT2) inhibitors (67) and glucagon-like peptide-1 receptor (GLP-1R) agonists (68) significantly improve systemic metabolic status. Whether their outstanding cardiovascular protective effects are partially achieved by systemically correcting the aberrant lactylation network within AS plaques is an extremely attractive direction for future research. It should be noted that when discussing these repurposed drugs, caution is warranted. Although existing studies suggest that these drugs may influence lactylation, direct causal evidence linking their cardiovascular benefits solely to lactylation modulation in human plaques is still emerging.

The diverse and promising therapeutic strategies targeting the protein lactylation network are systematically summarized in **Table 1**, which provides a clear overview of their target categories, specific targets, representative agents, key mechanisms, and potential outcomes in AS.

**Table 1:** Summary of potential therapeutic strategies targeting the protein lactylation network in atherosclerosis.

Target category	Specific target	Agent/drug	Mechanism & outcome in AS	References
Lactate Production	LDHA	FX-11	Inhibits LDHA activity, reducing lactate production to alleviate inflammation and delay plaque progression.	(53, 55)
		Oxamate		(54)
	HK	2-DG	Inhibits HK activity, blocking glycolytic flux to reduce lactate source.	(56)
	PDK	Dichloroacetate	Inhibits PDK activity, promoting pyruvate oxidation to indirectly reduce lactate.	(57)
Lactate Clearance	MCT4	VB124	Inhibits MCT4 function, increasing intracellular lactate to induce H3K18la and promote M2 polarization, thereby stabilizing plaques.	(29)
		Quercetin		(59)
		Diclofenac		(58)
Writer Enzymes	p300/CBP	C646	Inhibits p300/CBP lactyltransferase activity, blocking pathogenic lactylation-driven transcriptional programs.	(60)
		A-485		(61)
	AARS1	β-alanine	Inhibits AARS1 lactyltransferase activity, reducing global protein lactylation levels.	(62)
Eraser Enzymes	SIRT1	Resveratrol	Activates SIRT1 delactylase activity, downregulating lactylation levels to promote an anti-inflammatory phenotype.	(26)
	SIRT3	Honokiol	Activates SIRT3 delactylase activity, downregulating lactylation levels, showing protective effects.	(63)
Multi-target Agents	Lactylation-Associated Gene Network (e.g., PARP12, IRF8, NLRP3, etc.)	Ruanjian Qingmai Formula (TCM)	Multi-target regulation of this network inhibits macrophage pyroptosis/ferroptosis and reduces plaque burden.	(64)
Drug Repurposing	Lactate Metabolism Network	Metformin	Downregulates lactate generation and H3K18la, exerting anti-inflammatory effects.	(66)
	MCT1/4	Atorvastatin	May modulate lactate homeostasis by interfering with MCT function.	(65)
	Systemic Metabolism	SGLT2 inhibitors	Improves systemic metabolic status, potentially exerting cardiovascular protection by modulating the plaque lactylation network.	(67)
		GLP-1 receptor agonists		(68)

LDHA, lactate dehydrogenase A; HK, hexokinase; 2-DG, 2-deoxy-D-glucose; PDK, pyruvate dehydrogenase kinase; MCT4, monocarboxylate transporter 4; H3K18la, histone H3 lysine 18 lactylation; p300/CBP, p300/CREB-binding protein; AARS1, alanyl-tRNA synthetase 1; SIRT1, sirtuin 1; SIRT3, sirtuin 3; PARP12, poly(ADP-ribose) polymerase family member 12; IRF8, interferon regulatory factor 8; NLRP3, NOD-, LRR- and pyrin domain-containing protein 3; TCM, traditional Chinese medicine; SGLT2, sodium-glucose cotransporter 2; GLP-1R, glucagon-like peptide-1 receptor.

## 6 Summary and future perspectives

In summary, protein lactylation serves as a functional extension of the metabolite lactate at the molecular level, achieving a crucial coupling between metabolic reprogramming signals and immune responses within coronary AS plaques. Lactate, accumulated in the hypoxic and hyperglycolytic plaque microenvironment, precisely regulates key cellular processes by inducing lactylation modifications on critical transcription factors, metabolic enzymes, and histones. These processes include driving macrophage polarization toward an anti-inflammatory and reparative phenotype, participating in regulated cell death pathways such as ferroptosis and pyroptosis under specific conditions, and influencing homeostatic mechanisms like macrophage efferocytosis. These functional alterations profoundly impact the dynamic equilibrium of foam cell formation, lipid accumulation, and cellular clearance, thereby regulating plaque inflammatory status, the size and stability of the lipid-rich necrotic core. Furthermore, lactylation modifications affect platelet activation by altering the secretory profile of macrophages, ultimately modulating the risk of acute thrombotic events triggered by plaque rupture. Targeting this regulatory network allows direct intervention in plaque stability. Strategies such as modulating lactate generation, modifying enzyme activity, or affecting downstream effector proteins offer precise ways to influence plaque progression. This points toward a novel direction for the metabolic-targeted therapy of cardiovascular diseases.

However, this field is still in its infancy, and current understanding faces multiple limitations and controversies that require in-depth investigation. Most studies on the functional regulation of macrophages by protein lactylation and its therapeutic potential originate from models of other diseases such as cancer and sepsis, and their direct applicability and functional validation in the specific pathological context of AS remain insufficient. Although lactylation signals have been detected in AS plaques and correlated with macrophage infiltration, comprehensive establishment of its direct functional role and causality within the AS-specific pathological setting is still lacking. The majority of research relies on correlative analyses or *in vitro* lactate interventions, lacking direct evidence from specific manipulation of particular lactylation sites within the complex AS microenvironment to observe their impact on plaque phenotype. Furthermore, lactylation exhibits a seemingly paradoxical dual functionality within the plaque microenvironment: it participates in both pro-inflammatory and anti-inflammatory/repair processes; it can drive cell death while also influencing repair processes. This functional orientation is highly context dependent, likely influenced by the disease stage, local lactate concentration and exposure duration, heterogeneity of macrophage functional subsets, and complex interactions between lactylation and other modifications such as acetylation and methylation. Currently, a clear framework is lacking to predict how lactylation will dictate macrophage fate decisions in specific spatiotemporal contexts.

Concurrently, a series of fundamental research gaps and their underlying unresolved mechanisms remain to be addressed. It is still unknown whether macrophage functional subsets beyond the classical M1/M2 dichotomy (e.g., TREM2^hi^, proliferative subsets) possess their own specific lactylation modification networks and differential functional regulation. How the “crosstalk” between lactylation and other modifications such as acetylation, crotonylation, and methylation at identical or adjacent lysine residues integrates signals and determines the final output represents deep water in mechanistic research. Additionally, the spatiotemporal mechanisms by which the spatial lactate gradient within plaques (e.g., necrotic core edge vs. fibrous cap) differentially regulates lactylation modifications and functions of macrophages in different regions await elucidation.

Targeting the lactylation network for therapy also faces multiple challenges. The first is how to achieve cell- and site-specific intervention to avoid off-target effects from systemic modulation of lactate metabolism or pan-modifying enzyme activity. Second, the pharmacokinetics and durability of efficacy of drugs modulating lactate transport or modifying enzyme activity (e.g., MCT4 inhibitors, p300 inhibitors) within the complex AS microenvironment require evaluation. Furthermore, there is a need to identify biomarkers that reflect plaque lactylation activity or vulnerability to guide patient stratification and treatment monitoring.

Therefore, future research should be dedicated to achieving precise intervention, focusing on the following key directions: elucidating the dynamic regulatory principles of lactylation on macrophage function across different stages of AS, such as early and advanced phases; uncovering the spatiotemporal mechanisms by which the spatial lactate gradient within plaques (areas of high vs. low concentration) differentially regulates lactylation modifications and functions in macrophages; analyzing the specific lactylation networks and their impact on cell fate within macrophage functional subtypes that go beyond the classical M1/M2 dichotomy; and systematically clarifying the crosstalk between lactylation, acetylation, methylation, and other modifications in macrophages. Building on this foundation, efforts should aim to develop more specific targeting tools for lactylation modifications in atherosclerosis and to identify biomarkers associated with plaque vulnerability. These endeavors are crucial for driving the clinical translation of this mechanism and providing novel strategies for the precise prevention and treatment of AS.
